# *Candida auris* as an Emergent Public Health Problem: A Current Update on European Outbreaks and Cases

**DOI:** 10.3390/healthcare11030425

**Published:** 2023-02-02

**Authors:** Nicholas Geremia, Pierluigi Brugnaro, Maria Solinas, Claudio Scarparo, Sandro Panese

**Affiliations:** 1 Unit of Infectious Diseases, Department of Clinical Medicine, Ospedale “dell’Angelo”, 30174 Venice, Italy; 2 Unit of Infectious Diseases, Department of Clinical Medicine, Ospedale Civile “S.S. Giovanni e Paolo”, 30122 Venice, Italy; 3 Unit of Microbiology and Virology, Department of Medical Direction, Ospedale “dell’Angelo”, 30174 Venice, Italy

**Keywords:** *Candida auris*, emerging fungal disease, public health promotion, infectious disease prevention

## Abstract

*Candida auris* is considered to be an emerging fungal pathogen and is related to high mortality rates, persistent candidemia, inconsistencies in susceptibility testing results and misidentification by available commercial identification systems. Multidrug-resistant (MDR) and pandrug-resistant (PDR) strains are increasingly detected. In Europe, hospital outbreaks caused by *C. auris* have been reported in the United Kingdom (UK), Italy and Spain; however, several cases have been sporadically detected in all European countries. *C. auris* is difficult to control despite enhanced control measures due to its ability to survive for a long time in environments and colonize patients for prolonged periods. An adequate laboratory diagnostic capacity and national surveillance are fundamental to rapidly detect new *C. auris* cases and to apply the correct measures to circumscribe them and prevent their spread. Our narrative review aims to highlight the primary *C. auris* outbreaks and case reports that have occurred in Europe.

## 1. Introduction

From 2009, *Candida auris* has been considered to be a rising healthcare emergency worldwide. *C. auris* infections are related to high mortality rates, persistent candidemia, inconsistencies in susceptibility testing results and misidentification by available commercial identification systems. All this must be considered alongside a high risk of treatment failure, which complicates its management [1].

In 2009, *C. auris* was initially found in Japan [2,3]. However, a retrospective review of the *Candida* strain found *C. auris* in South Korea in 1996 [4]. Studies have suggested that *C. auris* emerged simultaneously and independently in four global regions (South Asia, East Asia, Africa and South America; also named clades I, II, III and IV, respectively). These four clades are genetically distinct [5]. Most recently, a new potential V clade was identified that was isolated from Iran [6]. In the last few years, *C. auris* infections have increased worldwide [1,7]. In many parts of Africa and Asia, *C. auris* is now considered to be endemic [8]. In addition, several outbreaks have been reported in European countries such as the United Kingdom (UK), Spain and Italy [1,8,9,10].

Multidrug-resistant (MDR) and pandrug-resistant (PDR) *C. auris* strains are increasingly detected worldwide. The most frequent resistance is to fluconazole (FLC), followed by amphotericin B (AMB) and voriconazole (VRC). Echinocandin remains the treatment of choice, but resistance can also affect this class of antifungal drugs [1,11].

The clinical presentation and the risk factors of a *C. auris* invasive infection are similar to other *Candida* infections. However, many studies have demonstrated the environmental persistence ability of *C. auris*, including in the air, on surfaces and bedding materials. Moreover, *C. auris* has been isolated from the skin of colonized patients (also during an effective antifungal treatment) for several months [1].

## 2. Identification

*C. auris* was first detected in the external ear canal of a 70-year-old Japanese woman. A 26S ribosomal DNA (rDNA) D1/D2 domain analysis, 18S internal transcribed spacer (ITS) rDNA region sequences and chemotaxonomic studies showed that the newly discovered *Candida* species (spp.) had a close phylogenetic relationship to the *Metschnikowiaceae* clade, particularly with *C. ruelliae* and *C. haemulonii* [3]. A retrospective study on historical Korean isolates revealed that *C. auris* strains were initially misidentified as *C. haemulonii* [12]. A genetic analysis based on ITS 1/2 and D1/D2 sequences showed that *C. auris* belongs to the *Metschnikowiaceae* family within the *Candida*/*Clavispora* clade such as *C. albicans*, *C. tropicalis*, *C. haemulonii* and *C. lusitaniae* [3].

**Figure 1:**
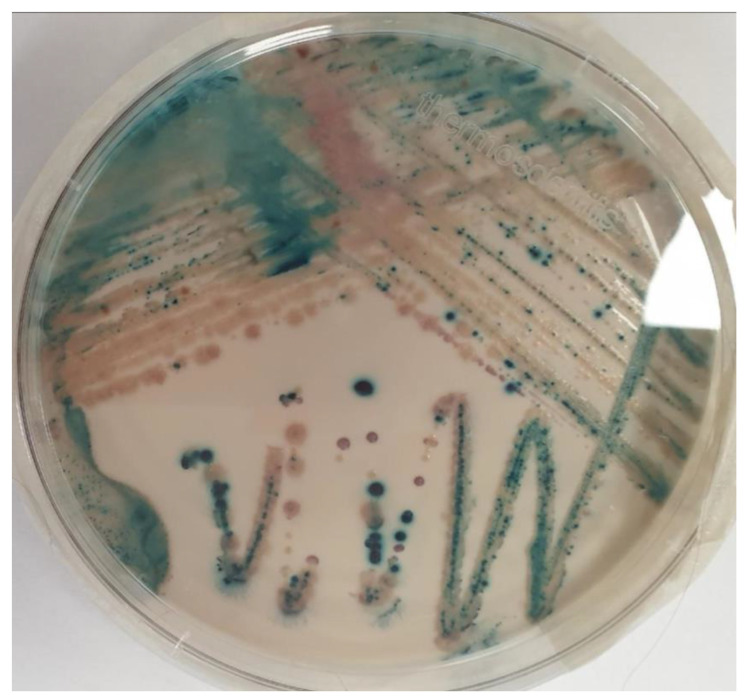
*Candida* isolates from Brilliance™ Candida Agar Base (Thermo Fisher Scientific^TM^, Waltham, MA, USA). *C. auris* (light blue with blue halo colonies), *C. krusei* (pink and fuzzy colonies) and *C. albicans* (green–blue colonies).

The misidentification of *C. auris* as another yeast species using conventional phenotypic and biochemical methods can be common (Table 1) [2,3]. The thermal tolerance property of growth at temperatures up to 42 °C on CHROMagarTM Candida Plus (CHROMagar, France) has been used to differentiate *C. auris* from other *Candida* spp. [13,14]. The diagnosis of *C. auris* infections includes biochemical-based tests such as analytical profile index strips, VITEK 2, BD Phoenix yeast identification and MicroScan. Nevertheless, these tests lack a comprehensive database for yeast identification [13]. Figure 1 shows *C. auris* identification.

The identification of yeasts by matrix-assisted laser desorption ionization-time of flight mass spectrometry (MALDI-TOF MS) analyses has the potential to quickly identify *C. auris*. However, initial attempts to identify *C. auris* using this tool were unsuccessful. Following this *C. auris* isolation across many countries, MALDI-TOF MS added isolates from all four major clades to their FDA-cleared databases [12,13]. In addition, DNA sequencing techniques such as polymerase chain reaction (PCR) have also been used for the identification of *C. auris*. For example, the PCR amplification of the D1/D2 region and ITS rDNA can be used to differentiate the principal phylogeographic clades of this species, but a further delineation of local hospital clusters required higher resolution methods, including amplified fragment length polymorphism (AFLP) and whole genome sequencing (WGS) analyses [14].

## 3. Virulence Factors

*C. auris* can express several virulence factors, including saps and lipases [15]. However, *C. auris* is less virulent than *C. albicans*. That characteristic was shown in murine and invertebrate *G. mellonella* infection models. In murine models, it was demonstrated that *C. auris* was much more virulent than *C. glabrata* and *C. haemulonii* [2,16]. This difference, compared with *C. albicans*, depended on the inability of *C. auris* to develop virulence factors such as hyphae or pseudohyphae, which play a critical role in tissue invasion [14]. Furthermore, *C. auris* is a haploid yeast whereas natural *C. albicans* isolates are diploid. This could have an essential role in the intrinsically low virulence of *C. auris*. In FLC-induced haploids, the *C. albicans* strain reduced their virulence compared with the diploid form [2,17]. The filamentous cells of *C. auris* are poorly implicated in its virulence during systemic infections, but could play a role in skin and environmental surface colonization [2].

## 4. Antifungal Resistance

FLC and echinocandins are the most used antifungal drugs to treat candidemia. Unfortunately, FLC (or other azole) resistance is common. A recent meta-analysis from Sekyere et al. showed that the most frequent resistance was to FLC (44.29%), followed by AMB (15.46%), VRC (12.67%), caspofungin (CAS) (3.48%), flucytosine (FC) (1.95%), itraconazole (ITZ) (1.81%), isavuconazole (ISA) (1.53%), posaconazole (POS) (1.39%), anidulafungin (AFG) (1.25%) and micafungin (MFG) (1.25%) [11,12]. MDR *C. auris* strains have been reported in several cases, showing resistance phenotypes to FLC and AMB [18]. Resistance to echinocandins is not so frequent. Chen et al. found that the resistance rates to CAS, MFG and AFG were 12.1%, 0.8% and 1.1%, respectively. However, almost all isolates resistant to CAS were from India (23.6%) [19].

The molecular mechanism for azole resistance in *C. auris* is mainly related to alterations in the lanosterol demethylase enzyme, which is encoded by the ERG11 gene. *C. auris* can also encode ATP-binding cassette (ABC) and major facilitator superfamily (MFS) efflux pumps, which are essential mechanisms of antifungal resistance, especially during the initial stages of biofilm development. When resistance to echinocandins; occur, it is due to mutations in FKS genes that encode a subunit of the β-D-glucan synthase. Moreover, changes to the cell membrane sterol and/or a given point mutation are potential mechanisms of AMB resistance [13,20].

Unfortunately, no antifungal susceptibility breakpoints for *C. auris* are currently standardized for the Clinical and Laboratory Standards Institute (CLSI) and European Committee on Antimicrobial Susceptibility Testing (EUCAST). Therefore, the Centers for Disease Control and Prevention (CDC) defined a *C. auris*-specific antifungal susceptibility interpretation based on a close phylogenetic relationship to other *Candida* spp. The correlation between the microbiologic breakpoints and clinical outcomes is not known. The current breakpoints are summarized in Table 1 [21].

**Table 1:** *C. auris*-specific antifungal susceptibility interpretation according to CDC [21].

Antifungal	MIC	Interpretation
Fluconazole	≥32	Isolates with MIC ≥ 32 were shown to have a mutation of the Erg11 gene
Voriconazole	NA	Consider using fluconazole susceptibility as a surrogate for other azoles. Occasionally, isolates that are resistant to fluconazole may respond to voriconazole
Amphotericin B	≥2	Isolates with a MIC of ≥2 should be considered to be resistant
Anidulafungin	≥4	Breakpoints are based on the distribution of echinocandin MICs of approximately 100 isolates from diverse geographic locations
Caspofungin	≥2	
Voriconazole	≥4	

MIC: minimum inhibitory concentration.

## 5. Risk Factors and Mortality Rates

Most *C. auris* cases have escalated within the last few years. The reported isolates were mainly isolated in males (64.76%). No reason has been given for the *C. auris* distribution by gender. Local variables and the health diversity of countries could play a role in the increase in *C. auris* male case rates. Patients with *C. auris* infections frequently presented several other underlying health comorbidities such as diabetes, sepsis, pulmonary diseases, bacterial pneumonia, renal diseases, transplants, immunosuppression, solid tumors, cardiovascular diseases, chronic otitis media and liver diseases [1].

The risk factors for *C. auris* infections are similar to other *Candida* spp. generic risk factors. Most frequently, infections occur in hospitalized patients, especially those admitted to the intensive care unit (ICU) or those who underwent surgery in the previous 30 days. Moreover, central venous catheters, hemodialysis catheters and permanent urinary catheters could be related to invasive *C. auris* infections [1,20,22].

Even with an appropriate antifungal treatment, invasive candidiasis has a mortality rate of up to 30–40%. Currently, there is limited information on specific *C. auris*-case fatality rates. However, several authors have suggested that the mortality rate of invasive *C. auris* infections is comparatively higher than that of *Candida* spp. For *C. auris*, the crude mortality rate was estimated to be 30% to 72% [1,18,23,24].

## 6. Case Reports and Outbreaks of *C. auris* in Europe

### 6.1. Italy

In mid-July 2019, the first *C. auris* case was detected at San Martino Hospital (Genoa) in a patient with no history of recent travel abroad, hospital admission or close contact with other *C. auris* cases [25]. Since then, new cases have sporadically been reported at the same hospital. From July 2019 to May 2020, *C. auris* was detected in 10 non-duplicate clinical isolates in the coronavirus disease-19 (COVID-19) ICU [9].

A subsequent increase in cases was observed throughout 2020 and 2021. In February 2022, 277 cases occurred at 8 healthcare facilities in Liguria and 11 patients were detected at facilities in the neighboring region of Emilia-Romagna. Most *C. auris* reported cases occurred at San Martino Hospital; only 67 cases were distributed to 7 other healthcare facilities in Liguria [26].

The *C. auris* isolates from the first cases at San Martino Hospital between 2019 and 2020 were closely related to the South Asian clade and all isolates, except one, originated from the same cluster of the index case [9]. Between July 2021 and March 2022, eight *C. auris* cases were observed at AOU Città della Salute e della Scienza (Turin). All the patients were admitted for critically ill conditions to the ICU. The majority (75%) had severe respiratory diseases due to COVID-19 and presented with prolonged hospitalizations, multiple complications and the simultaneous or previous presence of other infections, especially from difficult-to-treat Gram-negative microorganisms [27].

### 6.2. Spain

Between April and June 2016, four *C. auris* cases in continental Europe were identified at La Fe University Hospital and Polytechnic (Valencia). All of them were admitted to the post-surgical ICU [28]. From April 2016 to January 2017, 140 *C. auris* colonizations were identified at the same hospital and 41 of them developed invasive fungemia. All isolates were FLC- and VRC-resistant, but echinocandin- and AMB-susceptible. A phylogenic analysis revealed that the Spanish isolates were clonal, with an overall similarity of >96%. In addition, all Spanish isolates seemed to be genotypically connected to the South African isolates and one was grouped in the Venezuelan cluster [29].

In September 2017, *C. auris* was identified for the first time in the urine culture of a patient at the Consortium of the General University Hospital (Valencia). As a result, the patient was admitted to another hospital. A month later, on 14 October 2017, the second case was diagnosed. From September 2017 to August 2019, 203 patients were colonized and 30 invasive infections were diagnosed: 29 candidemia and 1 meningitis. All strains were resistant to FLC [30].

**Figure 2:**
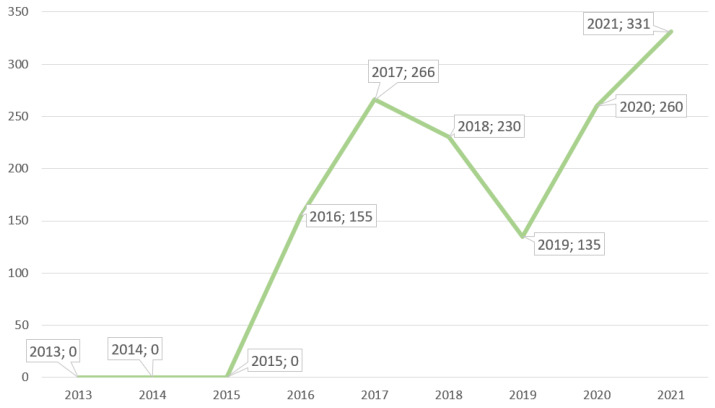
Spanish *C. auris* cases from 2013 to 2021 [33].

From the first Spanish *C. auris* case to 2019, Spain reported 786 cases of *C. auris* colonizations/infections, with a reduction in cases from 2017 to 2019 [10,31,32]. Unfortunately, during and after the COVID-19 pandemic, *C. auris* cases rose again (591 new cases from 2020 to 2021) [32]. Figure 2 shows the Spanish cases from 2016 to 2021.

Currently, Spain is the only European Union (EU) country where there is a reported regional endemicity [32].

### 6.3. The UK

The first three *C. auris* isolates were identified at the UK National Mycology Reference Laboratory (MRL) in 2013 from blood cultures from three unrelated patients from distant geographical localities. From 2013 to 2016, the MRL received 19 isolates from 6 hospitals [30,31].

Between June 2013 and March 2017, 225 *C. auris* cases were identified, with 61 infections (including 31 candidemia) across 22 hospitals. In addition, three significant outbreaks were reported in ICUs in London and Oxford [33,34].

The first hospital outbreak was in April 2015. *C*. *auris* was identified in a patient admitted to the medical-surgical adult ICU of Royal Brompton Hospital (London), a specialized cardiothoracic center. The yeast was initially cultured from a sternal wound. A total of 50 new *C. auris* cases were identified in the following 16 months from the index case. All isolates expressed a high-level FLC resistance. In most cases, *C. auris* exclusively colonized the skin or mucosa (56%). Only 9 patients had candidemia (16%). Nevertheless, 22 required an antifungal therapy. However, no deaths were directly attributed to infections by *C. auris* [35].

Phylogenetic analyses revealed that the UK isolates from the different outbreaks could be separated into three distinct clades, which contained isolates previously reported from India/Malaysia/Kuwait, South Africa and Japan/Korea. Furthermore, these data and the absence of *C. auris* in the UK before 2013 suggested multiple independent introductions of the yeast [34].

Between February 2015 and August 2017, after the identification of a case series of *C. auris* infections at the neuroscience ICU of Oxford University Hospitals (Oxford), Eyre et al. started an active screening period for *C. auris* colonization. Patients were tested on admission to the neuroscience ICU, weekly and on discharge. As a result, 70 patients were identified as being colonized and 7 had an invasive infection (4 candidemia and 3 central nervous system device-associated meningitis) [36].

In 2017, England provided a national prevalence study on admission screening for *C. auris* in ICUs. The study was conducted between May 2017 and April 2018. The study screened all ICU admissions who had no prior diagnosis of a *C. auris* colonization at eight adult hospitals. The results showed that *C. auris* screening for all 921 patients was negative. This finding demonstrated that in England, *C. auris* colonization among patients admitted to ICUs is rare [37].

Data from the UK showed that the early detection of outbreaks combined with isolation enhanced the infection control measures and screening could control the spread of *C. auris*. From January 2018 to May 2019, the UK reported  48 cases only [31,38].

### 6.4. Other European Countries

Between 2013 and 2017, the European Centre for Disease Prevention and Control (ECDC) *C. auris* survey also counted cases from Germany (7), France (2), Belgium (1) and Norway (1). Most patients were considered to be colonized; fungemia or other invasive infections were less frequent [39]. Excluding Spain, Italy and the UK, active *C. auris* surveillance between January 2018 and May 2019 detected *C. auris* cases from eight other countries, including Austria (1), France (1), Germany (3), Greece (1), the Netherlands (2), Belgium (1), Norway (1) and Poland (1) [26].

Favorable conditions for *C. auris* outbreaks in Europe were observed during the COVID-19 pandemic. The main reason for the spread of *C. auris* was an increase in the number of vulnerable patients requiring a high intensity of care combined with the concurrent overload of healthcare systems and the inability to maintain sufficient standards and contact precautions [40].

Sporadic cases were continuously identified scattered around Europe such as in Denmark, Switzerland, Finland, Ireland, Sweden and Russia [32,40,41,42,43]. In the period between 2019 and 2021, Denmark, France, Germany, Italy and Greece reported 14 *C. auris* outbreaks, defined as 2 or more cases with an epidemiological link [32].

In Greece, the first *C. auris* case was diagnosed in 2019 in a cystic fibrosis patient with no history of recent travel abroad or hospitalization [42]. Since the first identification, the trend of the cases of *C. auris* between 2020 and 2021 has been of particular concern. In 2021, 58 new cases were found, with a verified or plausible inter-facility spreading [32].

In France, a number of *C. auris* cases have been sporadically identified since 2015. To date, rare outbreaks with limited inter-facility spreading have been reported, with only 4 cases in 2020 and 4 cases in 2021 [32].

**Figure 3:**
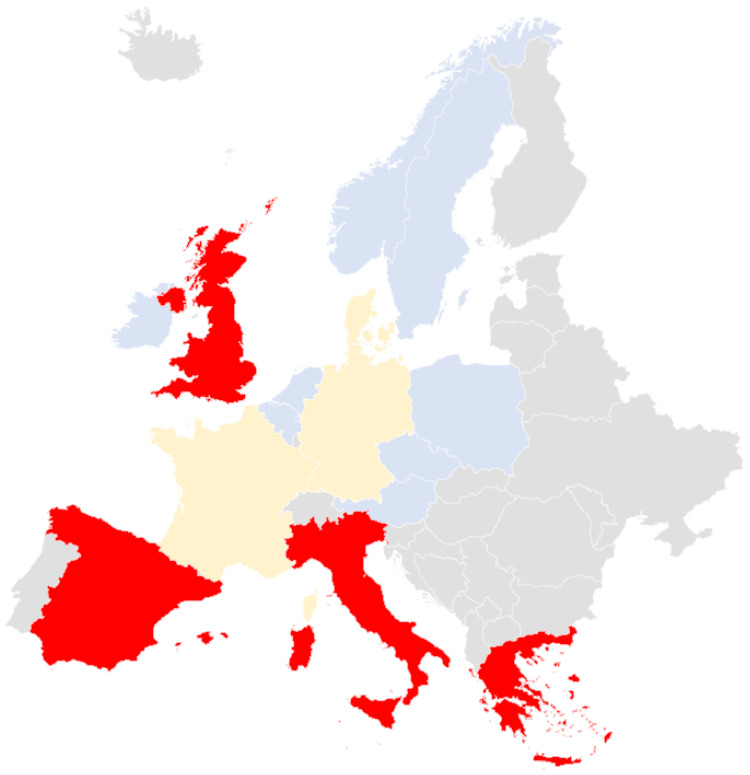
Principal cases of *C. auris* in Europe. In red: detected *C. auris* outbreak countries with inter-facility spreading or endemicity (Spain, Italy, Greece and the UK). In light yellow: country (Germany, France and Denmark) with sporadic outbreaks without or with only limited inter-facility spreading. In light blue: sporadic *C. auris* cases that were locally acquired or an unknown or imported origin [17,32,38].

In Germany, 27 *C. auris* cases have been reported, most of which occurred in patients who had recently been in contact with hospitals/healthcare providers abroad. However, the number of cases documented by the National Reference Center for Invasive Fungal Infections (NRZMyk) has rapidly increased [8,44]. Despite the increase in cases, Germany is an example of how the transmission of *C. auris* can be contained with prompt control measures [32]. Figure 3 shows the data on the *C. auris* case reports in Europe where *C. auris* was detected.

In the EU, between 2013 and 2021, there were 1812 identified *C. auris* cases (data not including the UK cases). A total of 44 (2.4%) cases were reported as imported and 10 (0.6%) as locally acquired. The origin was not always mentioned, but the most frequent countries of imported cases were Egypt, Ethiopia, Kenya, South Africa, Iraq, Kuwait, United Arab Emirates, India and Pakistan) [32].

## 7. Prevention Measures

If *C. auris* is isolated, robust actions are necessary to reduce the risk of developing hospital and regional outbreaks. A multidisciplinary approach is essential to implement infection prevention and control measures. A prompt identification and notification should trigger an investigation, including a detailed case review and a screening of close contacts. Infection control options include the application of contact precautions, single-room isolation or patient cohorting and, in a few cases, dedicated nursing staff. Screening of close contacts should be performed with axilla and groin swabs. Other samples (urine, wound swabs, blood cultures, etc.) can be collected to implement the diagnostic tools. The regular cleaning of surfaces with chlorine-based disinfectants (at a concentration of 1000 ppm), hydrogen peroxide or other disinfectants with a documented fungicidal activity is needed to reduce environmental contamination. Single-use or dedicated equipment should be preferred. The cleaning and disinfection of reusable equipment according to the manufacturer’s instructions should be ensured. The environmental sampling or screening of healthcare workers is not routinely recommended [38].

## 8. Discussion

Since its initial discovery, *C. auris* has been isolated in multiple areas of the world and its capability to determine healthcare outbreaks raises significant global concerns [45,46]. In the case of hospital outbreaks, the spreading of *C. auris* has been difficult to control, despite enhanced control measures. It is known that contaminated surfaces play an essential role as an environmental reservoir. Surveillance studies showed that *C. auris* could survive on surfaces for at least 14 days and on contaminated bedding for up to 7 days [46,47,48]. In the case of the colonization of patients, *C. auris* can be isolated on the skin for several months [46].

Additionally, healthcare workers could be responsible for *C. auris* spreading in the hospital environment. For example, an analysis performed during the first UK outbreak at Royal Brompton Hospital showed that the minimum contact time required to acquire *C. auris* was four hours [34]. In conclusion, infection control measures are fundamental to reduce the risk of developing hospital outbreaks. However, the necessity of implementing control measures such as contact precautions, single-room isolation or patient cohorting and dedicated nursing staff for patients who are colonized or infected appear to be non-demandable in the event of a single hospital *C. auris* case [38].

In Europe, hospital outbreaks caused by *C. auris* have occurred in the UK, Italy and Spain, but different case reports have been observed over almost all Europe. New *C. auris* cases were mainly diagnosed during the COVID-19 pandemic when the European healthcare system was subjected to continuous overload with an inability to maintain sufficient infection control measures and adequate antibiotic stewardship programs [37,45]. For example, during the COVID-19 pandemic, Greece evidenced an important rise in *C. auris* cases and outbreaks [32]. Following the constant increase in cases at the European level, countries must have an adequate laboratory capacity and national surveillance to detect *C. auris* cases. It should be remembered that commercially available laboratory tests used by clinical laboratories may not be able to identify or could misidentify *C. auris* strains (Table 2). As the UK data suggest, an early and immediate implementation of control measures such as alerts to healthcare staff, screening for carriage, contact tracing, enhanced infection prevention and control efforts increase the chances of containing *C. auris* cases [30,37,38].

In the case of hospital outbreaks, more sophisticated diagnostic tools such as AFLP and WGS are necessary to delineate the local yeast from spreading [14]. Unfortunately, not all clinical laboratories can perform these methods.

The majority of *C. auris* isolates are resistant to FLC, but a resistance to all three main antifungal classes and MDR fungal strains has also been described [9,10,16]. Although an antifungal therapy is a critical factor for therapeutic success, a recent meta-analysis showed no statistically significant association between mortality and resistance to FLC and AMB [18].

Currently, there is no evidence of a specific beneficial effect of antimicrobial stewardship on the emergence and spread of MDR *C. auris*. It is reasonable that the high use of broad-spectrum antibacterial and antifungal agents can play a role in the spread of MDR yeast such as *C. auris* [26].

In addition to the problem of antifungal resistance, there are currently no established breakpoints for the main available antifungal drugs. Unfortunately, the correlation between the microbiologic response and clinical outcomes is unknown. Tentative *C. auris*-specific antifungal susceptibility interpretations have been suggested by the CDC, but the problem is still open [20].

Although *C. auris* seems to have a strong propensity for patient-to-environmental-to-patient transmission in healthcare settings, its pathogenicity and invasive infection capacity are unknown [1,22,23]. As with other *Candida* spp., *C. auris* can cause infections, particularly in fragile patients who have prolonged hospitalizations or are recovering in ICUs. Invasive candidiasis has a mortality rate of up to 30–40%. However, due to the difficulty in discerning the real cause of death, it has been assumed that the mortality of *C. auris* may be around 70% [1,17,22,23,38]. Data from the CDC showed that patients with *C. auris* bloodstream infections had a 30-day mortality rate of 39% and a 90-day mortality rate of 58% [50]. High mortality rates were also reported in Venezuela (28%), India (50%) and Panama (78%) [51,52,53]. In a recent Italian study on *C. auris* invasive infections in critically ill patients, the 30-day mortality after the onset of *C. auris* candidemia was 26%. Notably, 4 out of 7 patients (57%) died within 30 days after the beginning of late recurrent candidemia [54]. In contrast, in the UK outbreak, no fatality could be directly attributed to *C. auris* [33,37]. In a meta-analysis of the global epidemiology and mortality of *C. auris*, the overall crude mortality ranged from 0 to 78%, with candidemia-associated mortality of 45% (vs. 21% in the non-candidemia group). A subanalysis of *C. auris* European mortality showed lower mortality rates (20%) [18].

## 9. Conclusions

Considering the potential of *C. auris* to generate hospital and inter-facility outbreaks, a local control protocol must be applied as soon as possible after the diagnosis of a new *C. auris* case. The possible laboratory misidentification of this yeast can delay the prompt application of all the measures that are necessary. Once *C. auris* has spread between facilities or regions, control is more difficult to achieve. For this reason, an adequate laboratory capacity for a correct fungal diagnosis is mandatory. This recommendation is valid, especially in the case of patients with a history of hospitalization in countries with a high *C. auris* prevalence. Currently, no antimicrobial stewardship program has evidenced the benefits of *C. auris* diffusion. A few European countries (Denmark and the UK) have shown that the spread of *C. auris* can be contained only with the planning of screening protocols, the isolation of suspected/confirmed cases and the application of additional hygiene measures.

In conclusion, the increase in European *C. auris* cases represents an important wake-up call for all Europe. We should prepare to be increasingly confronted with this potentially fatal new pathogen.

## Figures and Tables

**Table 2:** Commercial identification methods that can misidentify *C. auris* [49].

Identification Method	Organism *C. auris* Can Be Misidentified as
VITEK 2 YST *	*C. haemulonii* *C. duobushaemulonii*
API 20C	*Rhodotorula glutinis* *C. sake*
API ID 32C	*C. intermedia* *C. sake* *Saccharomyces kluyveri*
BD Phoenix yeast identification system	*C. haemulonii* *C. catenulata*
MicroScan	*C. famata* *C. guilliermondii* ** C. lusitaniae ** C. parapsilosis **
RapID Yeast Plus	*C. parapsilosis* **

* There are reports of *C. auris* being misidentified as *C. lusitaniae* and *C. famata* on VITEK 2. A confirmatory test may be necessary for these species. ** On cornmeal agar, *C. guilliermondii*, *C. lusitaniae* and *C. parapsilosis* generally make pseudohyphae; *C. auris* does not make hyphae or pseudohyphae instead. If hyphae or pseudohyphae are not present, any of these isolations should be submitted for further identification.

## Data Availability

Not applicable.
